# A Case of Prolonged Cholestatic Hepatitis Induced by Azithromycin in a Young Woman

**DOI:** 10.1155/2011/314231

**Published:** 2012-01-11

**Authors:** Caterina Maggioli, Luca Santi, Giacomo Zaccherini, Vittoria Bevilacqua, Francesca Giunchi, Paolo Caraceni

**Affiliations:** ^1^Department of Clinical Medicine, Alma Mater Studiorum University of Bologna, Policlinico Sant'Orsola, Via Albertoni 15, 40138 Bologna, Italy; ^2^Department of Internal Medicine, Geriatric and Renal Diseases, Alma Mater Studiorum University of Bologna, Policlinico Sant'Orsola, Via Albertoni 15, 40138 Bologna, Italy

## Abstract

Azithromycin, a semisynthetic macrolides, is frequently prescribed for the treatment of middle ear and upper respiratory tract infections, bronchitis, and community-acquired pneumonia. This antibiotic is usually well tolerated, and a rapid resolving cholestatic hepatitis has been described up to now only in six patients all, except one, over 65 years of age. We here report the case of a prolonged cholestatic hepatitis after administration of azithromycin in a young woman with no history of liver disease.

## 1. Introduction

Drug-induced liver injury (DILI) is an important cause of hepatic disease being estimated to account for approximately 5% of cases of jaundice and 10% of cases of acute hepatitis admitted to hospitals [1]. Antibiotics are an often underreported cause of hepatotoxicity occurring in about 1 of 10,000 individuals [2].

Azithromycin, an erythromycin semisynthetic derivative belonging to a subgroup of macrolides called azolides, is frequently prescribed for the treatment of middle ear and upper respiratory tract infections, bronchitis, and community-acquired pneumonia [3]. It is generally well tolerated, with less than 1% of patients discontinuing the medication because of adverse effects represented most often by gastrointestinal manifestations, including diarrhea, nausea, abdominal pain, and vomiting [4].

Contrary to erythromycin, which has been shown to induce cholestatic hepatotoxicity in 3.6/100,000 patients [5], azithromycin is a very rare cause of liver injury since, at the best of our knowledge, only five cases of symptomatic hepatitis have been described in adults [6–10]. In these patients, liver biopsy showed intrahepatic cholestasis and periportal necroinflammatory infiltrate with lymphocytes and eosinophils and liver injury rapidly resolved with discontinuation of the medication.

We here report the case of a prolonged cholestatic hepatitis after administration of azithromycin in a young woman with no history of liver disease.

## 2. Case Report

A 26-year-old woman was admitted to our hospital, a third-level center for the diagnosis and treatment of liver disease, at the end of June 2010 because of the persistence of jaundice in the last two months.

Her medical history included allergic asthma requiring seasonal cycles with inhaled corticosteroids and antihistaminics, intolerance to cephalosporins, and penicillins, ovarian cysts treated with ethinyl estradiol and cholecystectomy during infancy due to symptomatic biliary gallstones. She denied smoking, alcohol or herbal medicine consumption, illicit drugs use, or exposure to any toxic agent. Finally, she had no history of preexisting liver, cardiac, or renal disease.

At the beginning of May 2010, the patient started to assume azithromycin 500 mg daily per os prescribed by the general practitioner for an upper respiratory tract infection. At the third day of treatment, she presented a diffuse skin rash and conjunctivitis which rapidly resolved with dexamethasone and local tobramycin.

However, two days later, she developed jaundice and was then admitted to the local community hospital. Laboratory values showed marked hyperbilirubinemia (10.73 mg/dL, reference range <1.10 mg/dL), mostly conjugated (9.03 mg/dL, reference range <0.30 mg/dL), and a mild to moderate elevation of serum transaminases (AST 63 IU/l, normal values: <32 IU/l; ALT 125 IU/l, normal values: <31 IU/l) and cholestatic enzymes (gamma-GT 231 IU/l, normal values: <36 IU/l, ALP 445 IU/l, normal values: <280 IU/l). The serological tests for hepatitis A, B, and C viruses, Epstein-Barr (EBV) virus, and cytomegalovirus (CMV) were all negative. Immunological examination revealed normal immunoglobulin levels, but a positive antinuclear antibody (titer 1 : 80). Other abnormal lab tests revealed mild normocytic anemia (haemoglobin 11.5 g/dL, reference range >12 g/dL) and hypercholesterolemia (421 mg/dL, reference range <200 mg/dL). Her chest radiograph was normal. Abdominal ultrasound and magnetic resonance showed no morphological alterations of the liver, while the biliary tree was not dilated. The diagnosis of iatrogenic jaundice was made, and the patient was treated with prednisone (25 mg daily) and ursodeoxycholic acid (600 mg daily).

She was then discharged at the end of May without improvement of liver tests, which remained steadily elevated during the following month. The patient was therefore admitted to our hospital for a reevaluation of the liver disease.

At admission, the patient was jaundiced and presented hitching with multiple scratch marks over the entire body, particularly at the lower limbs. No other stigmata of chronic liver disease and liver failure were found. Her laboratory test values were as follows: total bilirubin 9.48 mg/dL, conjugated bilirubin 8.29 mg/dL, AST 103 IU/l, ALT 145 IU/l, gamma-GT 404 IU/l, ALP 523 IU/l, cholesterol 311 mg/dL and tryglicerides 395 mg/dL, white blood cells 9.290/mmc (normal values: 4.220–9.000/mmc), haemoglobin 12.4 g/dL, and platelets 455.000/mmc (normal values: 150.000–380.000/mmc). Renal function and electrolytes, thyroid hormones, pancreatic enzymes, C-reactive protein, serum albumin, and coagulative parameters were normal. Tests for hepatotropic viruses, including HBV-DNA, HCV-RNA, EBV-DNA, and CMV antigenemia, were negative. Metabolic diseases were also excluded as iron and copper metabolisms were normal. The immunological exploration revealed a positive antinuclear antibody (ANA) with a speckled pattern and positive anti-SS-A and SS-B antibodies, but immunoglobulines levels and anti-liver-kidney microsome (anti-LKM), anti-smooth muscle (anti-SMA), anti-mitochondrial (anti-AMA), anti-locus ceruleus (anti-LC), anti-DNA, anticardiolipin, antitransglutaminase (anti-TG), and antineutrophil cytoplasmic (ANCA) antibodies were all negative.

**Figure 1:**
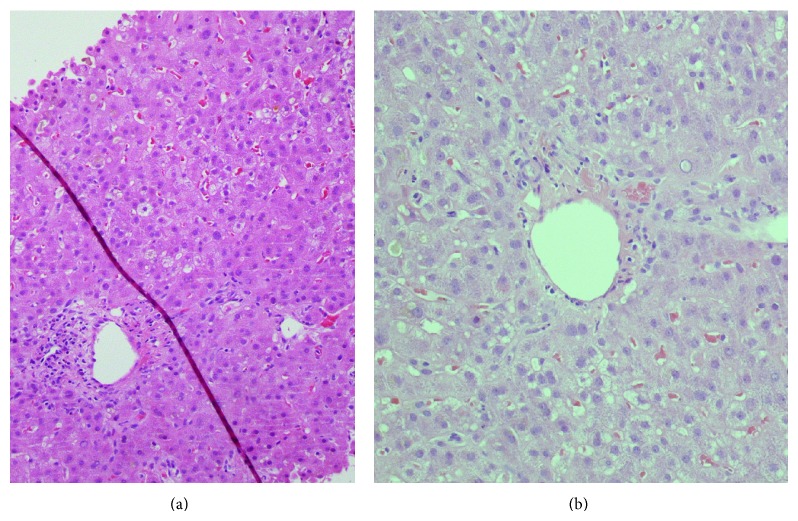
Representative pictures of liver histology at low (a) and high (b) magnification. Description in the text.

An abdominal ultrasound was repeated confirming the absence of alterations of the liver parenchyma and biliary ducts. Subsequently, she had a liver biopsy that revealed a picture of cholestatic hepatitis with a severe reduction of the native biliary ducts. More specifically, histology showed a preserved liver architecture with fibrous expansion of portal tracts (Figure 1). The portal tracts presented a severe reduction in the number and lumen diameter of bile ducts and mild chronic inflammatory infiltrate with plasma cells and neutrophils and eosinophils. Common bile thrombi, with foamy macrophages, common aspects of hepatocyte rosettes, rare granulomas, hepatocyte ballooning degeneration, and hepatocyte pleomorphism, glycogen nuclei, rare Councilman bodies, and necroinflammatory foci were found within the lobule parenchyma.

 According to the clinical scale for the diagnosis of DILI developed and validated by Maria and Victorino [1], we confirmed the initial diagnosis of azithromycin-induced liver injury. Taking into account the absence of extrahepatic manifestation, except for the initial transient skin rash, and of a positive rechallenge test, our patient reached a total score of 15 corresponding to a “probable” diagnosis of DILI. However, an important information came up from a more accurate anamnesis: an episode of transient dark urine and hypocholic stools following a 3-day treatment with azithromycin, given for an upper respiratory tract infection, occurred few months before. Thus, if this episode represented the first exposure to the drug, the actual jaundice could be considered the positive rechallenge test. As a result, the patient reached 18 as total score corresponding to a “definite” diagnosis of DILI.

During hospitalization, the patient was treated with ursodeoxycholic acid, vitamin E, glutathione, and progressively decreasing doses of prednisone without obtaining a significant improvement of the lab tests.

The patient was discharged after 9 days of hospitalization and followed in the outpatient clinic. Her laboratory tests progressively decreased and all returned within the normal ranges after approximately one year from the onset of jaundice (Table 1). At the last visit in October 2011, she was in good clinical conditions with normal levels of bilirubin and liver enzymes.

## 3. Discussion

Erythromycin, a naturally occurring macrolide, and its derivatives, clarithromycin and azithromycin, are frequently used antibiotics in general practice. While the use of erythromycin carries an established, albeit low, risk of liver injury, hepatotoxicity appears to be negligible with azithromycin [3].

At the best of our knowledge, only few cases of azithromycin-induced cholestatic hepatitis have been reported [6–11]. All the cases in adult individuals (>18 years of age) [6–10] show common clinical features. Symptoms, usually represented by jaundice and hitching, developed few days (from 3 to 10) after the beginning of azithromycin therapy, and their resolution occurred within a maximum of two months (from 4 to 60 days) after antibiotic withdrawal. Except one, all patients aged over 65 years, and liver histology, when available, showed intrahepatic cholestasis and periportal necroinflammatory infiltrate with lymphocytes and eosinophils.

Our patient is similar to those described in the previous reports with regard to the early onset of symptoms after drug exposure, the prevalent cholestatic pattern of liver injury, and the full recovery. However, she differs for the young age, the quite longer persistence of jaundice, and the very slow normalization of liver tests which occurred after almost a year. The delayed improvement was likely the result of the severity of the histological lesions characterized by a severe biliary ductopenia. Thus, our case revealed the possibility that azythromicin may cause severe liver injury potentially able to induce a vanishing bile duct syndrome.

DILI is typically idiosyncratic and is usually unpredictable until the drug is given. Mechanisms of hepatotoxicity include individual genetic variations in the drug metabolism and immune reactions to the drug or its metabolites [1, 12]. Azithromycin reaches hepatic concentrations exceeding those measured in serum by 25- to 200-folds due to its long half-life (68 hours) and its extensive uptake by the liver [13]. Hepatotoxicity, which appears to be unrelated to the administered dose, is likely favoured by the high tissue levels of the drug or its metabolites. Because liver damage in all the cases reported develops within very few days from drug exposure, the effect of azithromycin appears to be intrinsic.

DILI is often misdiagnosed since a correct medication history showing a temporal relationship between drug administration and onset of liver injury is frequently difficult to ascertain. Furthermore, drugs can mimic almost every naturally occurring liver disease affecting man, and, finally, all the other potential causes of liver disease need to be ruled out.

As for the only other reported case of azithromycin-induced cholestatic hepatitis in a young adult [7], our patient had a positive history of oral contraceptives use. However, the causative role of estrogens was excluded in this patient because she stopped their assumption several months before DILI following a prolonged and continuous assumption for years. Although estrogen-induced cholestasis is well accepted [14], its occurrence is now becoming progressively less frequent as a result of the decreasing hormone content in the contraceptive preparation.

We are also quite confident to have excluded any other liver disease by clinical, laboratoristic, imaging, and histological data. Although the positivity for ANA and anti-SS-A and anti-SS-B indicated the possibility of a differential diagnosis with autoimmune hepatitis, the absence of clinical manifestations of other immunological diseases and the lack of response during treatment with steroids favoured the diagnosis of DILI which was confirmed by the spontaneous normalization of the liver lab tests in the following months.

In conclusion, practitioners should be aware that azithromycin is able to induce DILI, including a prolonged symptomatic course of cholestatic hepatitis, in order to recognize early an adverse reaction to the drug thus leading to its immediate withdrawal and avoiding a repeated future exposure.

##  Conflict of Interests

All authors declare no conflict of interests.

## Figures and Tables

**Table 1:** Time course of liver function tests. ALT: alanine transaminase; AST: aspartate aminotransferase; ALP: alkaline phosphatase; G-GT: gamma-glutamyl transpeptidase; n.v.: normal values.

	21/05 1st Hospital admission	14/06 Discharge	28/06 2nd Hospital admission	14/08 Followup	24/08 Followup	8/09 Followup	15/12 Followup	7/4/11 Followup
ALT (n.v.: <31 IU/L)	125	137	145	313	312	228	150	15
AST (n.v.: <32 IU/L)	63	71	103	135	135	107	96	18
Total bilirubin (n.v.: <1.1 mg/dL)	10.7	11.6	9.48	6.02	4.42	3.12	1.97	0.26
ALP (n.v.: <280 IU/L)	445	140	523	426	418	371	320	73
G-GT (n.v.: <36 IU/L)	231	105	404	1912	1890	1369	256	19
